# Correlates of Lifetime Physical Abuse Among Schoolchildren Aged 15 Years in Post-communist Albania

**DOI:** 10.3389/fpubh.2021.607493

**Published:** 2021-07-28

**Authors:** Gentiana Qirjako, Qamil Dika, Iris Mone, Xheladin Draçini, Loreta Kuneshka, Enver Roshi, Genc Burazeri

**Affiliations:** ^1^Faculty of Medicine, University of Medicine, Tirana, Albania; ^2^Department of Sports Medicine, University of Sports, Tirana, Albania; ^3^Department of International Health, School CAPHRI (Care and Public Health Research Institute), Maastricht University, Maastricht, Netherlands

**Keywords:** Albania, child abuse, correlates, HBSC, lifetime physical abuse, schoolchildren

## Abstract

**Aim:** Our aim was to assess the prevalence and correlates of lifetime physical abuse among schoolchildren in Albania, a post-communist country in South Eastern Europe which is currently undergoing a rapid socioeconomic transition.

**Methods:** The third wave of Health Behavior in School-Aged Children (HBSC) in Albania was conducted in 2017–18 including a nationwide representative sample of 1,708 schoolchildren aged 15 years (54% girls; response rate: 95%). Children were asked to report on lifetime physical abuse and a wide range of socio-demographic factors, lifestyle factors and health status characteristics. Binary logistic regression was used to assess the independent association of lifetime physical abuse with covariates.

**Results:** Overall, the prevalence of lifetime physical abuse was about 32% (30% in boys vs. 32% in girls). In multivariable-adjusted logistic regression models, independent positive correlates of lifetime physical abuse among Albanian schoolchildren included lifetime smoking (OR = 1.5, 95% CI = 1.1–2.2), lifetime alcohol consumption (OR = 1.6, 95%CI = 1.2–2.1), irritability (OR_[dailyvs.rarely/never]_ = 2.0, 95%CI = 1.3–3.0), and especially lifetime witnessed domestic violence (OR = 4.2, 95%CI = 2.2–7.9). Conversely, a higher score on life satisfaction was inversely related to lifetime physical abuse (*P* < 0.01).

**Conclusion:** Our study provides novel evidence about the magnitude and selected independent correlates of lifetime physical abuse among schoolchildren in Albania, a country still embedded in an everlasting transition which is associated with tremendous changes in family structure, community links and societal norms and values. Irrespective of a wide range of sociodemographic factors and health characteristics, lifetime smoking, alcohol consumption, irritability, a lower score on life satisfaction and, particularly, witnessed domestic violence were strong and significant correlates of lifetime physical abuse among Albanian schoolchildren aged 15 years.

## Introduction

According to the World Health Organization (WHO): “*Child maltreatment is defined as the abuse and neglect that occurs to children under 18 years of age. It includes all types of physical and/or emotional ill treatment, sexual abuse, neglect, negligence and commercial or other exploitation that results in actual or potential harm to the child's health, survival, development or dignity in the context of a relationship of responsibility, trust or power”* (1, 2).

Conferring to a WHO landmark report, the estimated prevalence of child maltreatment ranges from 5 to 50% (3). However, physical abuse in particular, which is on the focus of this article, has been reported as high as 65% in some countries (4). Regarding the European region, according to a meta-analysis, the prevalence of physical abuse was estimated at about 23% (5). Furthermore, it has been reported that 850 European children <15 years die each year from child maltreatment (6).

Immediate medical and psychological trauma aside, child maltreatment has numerous deleterious lifetime effects (1). Hence, it has been convincingly shown that individuals who have experienced child maltreatment are at increased risk of suicide (7), repeated maltreatment or victimization by others (1, 7), risky sexual behaviors (8), substance abuse (9), poor physical and mental health (7, 10), in addition to a higher risk for violence perpetration and criminal behavior (1, 7). Furthermore, child maltreatment has a negative economic impact due to expenditure on health care, education and social services, and through productivity losses over the victim's lifetime (1, 11). However, the evidence on the extent and independent predictors of child maltreatment in European countries is scarce, particularly in countries of South Eastern Europe (3, 6). Identification of independent risk factors of child abuse would be relevant for researchers, policymakers and decision-makers, and especially valuable for practitioners involved in child protection and other child care services.

The “Health Behaviors in School-Aged Children” (HBSC) is an international study of the WHO Regional Office for Europe, which has started in 1983–84 in a few countries, but currently includes 50 countries and regions across Europe and North America (12). This survey, carried out every four years and funded at national level in each country, employs an international-prototype self-report questionnaire aiming at assessing adolescent health and well-being (13).

Albania became part of the network of countries participating in the HBSC study since 2007. In 2009, Albania participated for the first time in the HBSC 2009–10 study. To date, three rounds of HBSC survey have been implemented in Albania (the second round in 2013–14, and the third round in 2017–18). The internationally standardized HBSC questionnaire has undergone some minor adjustments related to the health-educational specificities of Albania.

The information on the prevalence and determinants of child abuse in Albania is scarce. Albania has been described as a patriarchal society (14, 15) and the available evidence points to a high prevalence of intimate partner violence (16). Child abuse and other forms of domestic violence constitute an under-researched topic in former European countries, particularly in the Western Balkan countries including Albania (17).

In this context, based on the recent HBSC round conducted in 2017–18, the aim of this study was to assess the prevalence and correlates of lifetime physical abuse among schoolchildren in Albania, a post-communist country in South Eastern Europe which is currently undergoing a rapid socioeconomic transition associated with social disruption and increasing rates of domestic violence (17). In the absence of prior studies on child physical abuse in Albania, we hypothesized an association with unhealthy lifestyle characteristics including smoking and alcohol consumption. To the best of our knowledge, this is one of the very few studies assessing the independent correlates of child physical abuse in the transitional Western Balkan countries.

## Methods

The third wave of HBSC in Albania was conducted in 2017–18. HBSC is cross-sectional study based on an internationally standardized instrument (13) which has been validated in the Albanian context before implementing the first round in 2009–10.

### Study Population

Based on the HBSC international protocol, the minimum sample size needed for each age group is 1550 pupils (13). In Albania, the targeted study population consisted of a nationwide representative sample of schoolchildren aged 15 years (stratified multistage cluster sampling with probability proportional to size [PPS]). Stratification was based on prefectures of Albania, in both rural areas and urban areas of the country. Of note, the population size is more or less equal in urban vs. rural areas of the country. Such a stratification was used in order to ensure a fair representativeness of the sample at a national level. Based on these considerations, a nationwide sample of 1796 schoolchildren aged 15 years was drawn (961 girls, or 53.5%). Of 1796 targeted individuals, 88 schoolchildren refused to participate in the survey and/or provided considerably incomplete or non-valid information (43 girls and 45 boys). Hence, the study sample included 1708 schoolchildren aged 15 years (918 girls, or 53.7% of the overall sample). The response rate was 1708/1796 = 95.1%.

### Data Collection

A structured self-administered questionnaire included information on lifetime physical abuse and a wide range of socio-demographic factors, lifestyle factors and health status characteristics.

*Lifetime physical abuse:* children were asked whether a parent or other adult in the household had ever hit, beaten, kicked or physically tried to hurt them in any way. In the analysis lifetime physical abuse was dichotomized into: yes vs. no.

*Correlates:* socio-demographic characteristics included sex of schoolchildren (boys vs. girls), mother's and father's current employment status (dichotomized into: yes vs. no), and family income (dichotomized in the analysis into: poor vs. not poor). Lifestyle factors included lifetime smoking (yes vs. no), lifetime alcohol consumption (yes vs. no), lifetime use of cannabis (yes vs. no), frequency of physical activity in the past week preceding the survey (0, 1–2, 3–4, and 5–7 days), and lifetime sexual relationships (yes vs. no). Furthermore, schoolchildren were asked about their self-perceived health status (excellent, good, fair, poor), frequency of irritability (about every day, more than once a week, about every week, rarely or never), life satisfaction (in a range from 0 [worst possible] to 10 [best possible]), and self-reported height (in cm) and weight (in kg) [based on which, the body mass index (BMI) was calculated]. In addition, schoolchildren were asked whether they had seen or heard one of their parents/carers being slapped, kicked, punched, beaten or deliberately hurt by a partner or ex-partner in their homes (referred to as “witnessed domestic violence” and dichotomized in the analysis into: yes vs. no).

All schoolchildren were informed about the aim and objectives of the study and were explained in sufficient details the particular aspects related to the anonymity of the survey. The study was approved by the Scientific Committee of the national Institute of Public Health in Albania (in 2017) and the data collection process was also confirmed by the Ministry of Education, Sport and Youth (in 2018).

### Statistical Analysis

Overall, 71 schoolchildren provided partial data for the lifetime physical abuse and further 96 participants provided partial information for some of the covariates included in this analysis. These 167 individuals (71 + 96) were excluded from the analysis. The remaining sample size was: 1708–167 = 1541. Therefore, the final response rate of the sample included in the statistical analysis was: 1541/1708 = 90.2%.

Fisher's exact test was used to compare the distribution of lifetime physical abuse (no vs. yes) according to different categories (Table 1) of sociodemographic characteristics (sex, parental employment status, and family income), lifestyle factors (physical activity, and lifetime: smoking, alcohol consumption, cannabis and sexual relationships), witnessed domestic violence, self-perceived health, and irritability of schoolchildren.

**Table 1:** Distribution of lifetime physical abuse by socio-demographic characteristics, lifestyle factors and health status of Albanian schoolchildren aged 15 years, HBSC 2017–18.

**Categorical variables**	**Total**	**No**	**Yes**	***P*-value**^†^****
**Gender:**				
Boys	746 (100.0)^*^	520 (69.7)	226 (30.3)	0.364
Girls	891 (100.0)	602 (67.6)	289 (32.4)	
*Total*	*1637 (100.0)*	*1122 (68.5)*	*515 (31.5)*	
**Father's employment:**				
Yes	1418 (100.0)	974 (68.7)	444 (31.3)	0.745
No	200 (100.0)	135 (67.5)	65 (32.5)	
**Mother's employment:**				
Yes	960 (100.0)	648 (67.5)	312 (32.5)	0.513
No	652 (100.0)	451 (69.2)	201 (30.8)	
**Family income:**				
Not poor	1300 (100.0)	893 (68.7)	407 (31.3)	0.640
Poor	324 (100.0)	218 (67.3)	106 (32.7)	
**Smoking:**				
No	1309 (100.0)	951 (72.7)	358 (27.3)	< 0.001
Yes	311 (100.0)	163 (52.4)	148 (47.6)	
**Alcohol consumption:**				
No	1034 (100.0)	776 (75.0)	258 (25.0)	< 0.001
Yes	582 (100.0)	333 (57.2)	249 (42.8)	
**Cannabis use:**				
No	1530 (100.0)	1059 (69.2)	471 (30.8)	0.019
Yes	80 (100.0)	45 (56.3)	35 (43.8)	
**Physical activity:**				
0 days/week	64 (100.0)	37 (57.8)	27 (42.2)	0.265
1–2 days/week	381 (100.0)	259 (68.0)	122 (32.0)	
3–4 days/week	554 (100.0)	381 (68.8)	173 (31.2)	
5–7 days/week	607 (100.0)	424 (69.9)	183 (30.1)	
**Sexual relationship:**				
Yes	321 (100.0)	211 (65.7)	110 (34.3)	0.313
No	1220 (100.0)	839 (68.8)	381 (31.2)	
**Witnessed violence:**				
No	1518 (100.0)	1069 (70.4)	449 (29.6)	< 0.001
Yes	82 (100.0)	27 (32.9)	55 (67.1)	
**Health status:**				
Excellent	1113 (100.0)	807 (72.5)	306 (27.5)	< 0.001
Good	433 (100.0)	265 (61.2)	168 (38.8)	
Fair	77 (100.0)	43 (55.8)	34 (44.2)	
Poor	11 (100.0)	6 (54.5)	5 (45.5)	
**Irritability:**				
About every day	232 (100.0)	129 (55.6)	103 (44.4)	< 0.001
More than once a week	229 (100.0)	137 (59.8)	92 (40.2)	
About every week	265 (100.0)	156 (58.9)	109 (41.1)	
About every month	347 (100.0)	249 (71.8)	98 (28.2)	
Rarely or never	508 (100.0)	410 (80.7)	98 (19.3)	
**Numerical variables**	**Total**	**No**	**Yes**	***P*** **-value** ^**¶**^
**Height** ***(cm)***	168.7 ± 9.0^‡^	168.9 ± 8.8	168.3 ± 9.1	0.195
**Weight** ***(kg)***	58.6 ± 10.6	58.6 ± 10.6	58.5 ± 10.4	0.992
**BMI** ***(kg/m**^**2**^**)***	20.5 ± 3.1	20.5 ± 3.0	20.6 ± 3.0	0.413
**Life satisfaction** ***(range from 0 [worst] to 10 [best])***	7.8 ± 2.0	8.0 ± 1.9	7.4 ± 2.1	< 0.001

* *Absolute number and row percentages (in parentheses). Discrepancies in the totals are due to missing covariate values*.

† *P-values from Fisher's exact test*.

‡ *Mean values ± standard deviations*.

¶ *P-values from Mann-Whitney U-test*.

*Italic values refer to the “total values”*.

Conversely, Mann-Whitney U-test was employed to compare mean values of BMI and life satisfaction between schoolchildren who reported lifetime physical abuse and those who did not have such an experience ever.

Binary logistic regression was used to assess the independent association of lifetime physical abuse with covariates (sociodemographic factors, lifestyle factors including BMI, witnessed domestic violence, self-perceived health, irritability, and life satisfaction). All variables (Table 2) were entered into the logistic regression models in a backward stepwise elimination procedure with a *p*-value to exit set at *P* > 0.10. Multivariable-adjusted odds ratios (ORs), 95% confidence intervals (95%CIs) and *p*-values were calculated. The analyses met the goodness-of-fit criterion as appraised by the Hosmer-Lemeshow test (18).

**Table 2:** Independent correlates of lifetime physical abuse among Albanian schoolchildren aged 15 years, HBSC 2017–18.

	OR^*^	95%CI^*^	P^*^
**Smoking:**			
Yes	1.00	Reference	0.019
No	1.52	1.07–2.16	
**Alcohol consumption:**			
Yes	1.00	Reference	0.002
No	1.56	1.17–2.08	
**Witnessed violence:**			
Yes	1.00	Reference	<0.001
No	4.18	2.21–7.88	
**Life satisfaction**	0.90	0.85–0.96	0.002
**Irritability:**			** <0.001 (4)** **^†^**
About every day	2.02	1.34–3.03	0.001
More than once a week	2.05	1.39–3.04	<0.001
About every week	2.14	1.47–3.13	<0.001
About every month	1.16	0.79–1.68	0.451
Rarely or never	1.00	Reference	-

* *Odds Ratios (OR: lifetime physical abuse vs. no abuse), 95% confidence intervals (95%CIs) and p-values (P) from binary logistic regression. The following variables were entered in a backward stepwise elimination procedure with a p-value to exit set at P > 0.10: gender, father's employment, mother's employment, family income, smoking, alcohol consumption, cannabis use, physical activity, BMI, sexual relationship, witnessed violence, health status, life satisfaction and irritability. Only the variables retained in the final logistic regression models are presented in the table*.

† *Overall p-value and degrees of freedom (in parenthesis)*.

For all statistical tests employed, a *p*-value of ≤ 0.05 was considered as statistically significant.

Statistical Package for Social Sciences (SPSS, version 19.0) was used for all the statistical analyses.

## Results

Table 1 presents the distribution of lifetime physical abuse by selected socio-demographic factors, lifestyle characteristics and health status of the Albanian children aged 15 years included in the HBSC 2017–18 survey.

The overall prevalence of lifetime physical abuse in this nationwide representative sample of Albanian schoolchildren was about 32%. There was no evidence of sex-difference (30% in boys vs. 32% in girls). Also, there were no significant relationships with the socioeconomic factors (parental employment status and family income). Conversely, the prevalence of lifetime physical abuse was considerably higher in children who reported lifetime smoking compared with those who had never smoked in their lives (48 vs. 27%, respectively; *P* < 0.01). Similarly, the prevalence of lifetime physical abuse was substantially higher in children who reported lifetime alcohol consumption compared with their counterparts who had never consumed alcohol in their lives (43 vs. 25%, respectively; *P* < 0.01). Furthermore, the prevalence of lifetime physical abuse was higher among children who reported lifetime cannabis use than in those who had never used cannabis (44 vs. 31%, respectively; *P* = 0.02). Physical exercise and BMI were not significantly related to lifetime physical abuse in children. On the other hand, there was a graded inverse association of better self-perceived health status, higher scores on life satisfaction and lack of irritability with lifetime physical abuse among children (all *P* < 0.01). There was no association with lifetime sexual relationships, but a remarkably strong association between lifetime physical abuse and lifetime witnessing of domestic violence: 67% of children who reported witnessing domestic violence had been also subject to physical abuse compared with only 30% of children who had never witnessed domestic violence in their lifetime (Table 1).

Table 2 presents the independent association of lifetime physical abuse in children, with all covariates entered into the logistic regression models in a backward stepwise elimination fashion. Upon adjustment for all covariates, independent positive correlates of lifetime physical abuse among Albanian schoolchildren included: lifetime smoking (OR = 1.5, 95%CI = 1.1–2.2); lifetime alcohol consumption (OR = 1.6, 95%CI=1.2–2.1); irritability (OR_[daily vs. rarely/never]_ = 2.0, 95%CI = 1.3–3.0); and, especially, the lifetime witnessing of domestic violence (OR = 4.2, 95%CI = 2.2–7.9). Conversely, life satisfaction was inversely related to lifetime physical abuse in children (OR_[for an increment of 1 unit in a score ranging from 0−10]_) = 0.9, 95%CI = 0.8–1.0).

## Discussion

### Main Findings

Main findings of this study include a high prevalence of lifetime physical abuse among schoolchildren aged 15 years in a transitional post-communist country in South Eastern Europe. Hence, almost one in three schoolchildren had ever experienced physical abuse by a parent or other adult in the household. Irrespective of a wide array of sociodemographic characteristics, lifestyle factors and health status, lifetime smoking, alcohol consumption, irritability, a lower score on life satisfaction and, particularly, witnessed domestic violence were strong and significant correlates of lifetime physical abuse among Albanian schoolchildren aged 15 years.

### Comparison With Other Studies

A fairly recent report based on three consecutive surveys conducted in Poland, another post-communist country in Central Europe, estimated the prevalence of physical violence among schoolchildren as follows: in rural areas 17.1%; in urban-rural areas 20.7%; in urban areas 29.4% (19). More specifically, acts of physical violence expressed in the form of single or repeated slapping with the hand and/or kicking, and the like, were reported by 11.3% of respondents from the rural community (2017 survey); in the urban-rural community it was 19.7% (2016 survey); whereas in the urban community it was 24.2% (2015 survey) (19). In our study, we did not observe any significant urban-rural differences. Nevertheless, on the whole, the prevalence estimates from our study conducted in Albania (32%) are higher than in Poland.

However, physical abuse has been reported as high as 65% in some African countries (4), an estimate which is twice as high compared with the findings of the current study carried out in post-communist Albania.

Of note, child physical abuse is a serious issue also for the industrialized countries. Hence, in Germany, in 2016, there were registered 3,621 victims of physical abuse among children, and further 11,777 non-fatal cases of child physical abuse, in a population of approximately 13.5 million children and adolescents <18 years residing in Germany in 2016 (20). Furthermore, three surveys conducted in Germany in the past decade have reported a prevalence of 12–13% for child physical abuse (21–23), which is significantly lower compared to our study conducted in Albania.

According to a meta-analysis, the prevalence of physical abuse in the European region was estimated at about 23% (5), which is also lower than our finding (32%). Hence, seemingly, the prevalence of child physical abuse in transitional Albania is higher than the European average and one of the highest in the Eastern European region when compared with e.g. Romania (about 23%), Montenegro (20%) and especially the North Macedonia (only 7%) (24).

Our finding on the prevalence of child physical abuse goes in line with the description of Albania as a patriarchal society (14, 15) and also with some previous evidence from this country indicating a high prevalence of intimate partner violence (16).

### Study Limitations

Nevertheless, this study conducted in Albania may have several limitations including the possibility of selection bias, information bias, and its cross-sectional design. On the whole, the response rate of this study was very high (95%), but 88 schoolchildren refused to participate and/or provided no valid information. In addition, findings from this study can be generalized to all Albanian schoolchildren aged 15 years, but not to children of the same age who do not attend school. On the other hand, the instrument for assessment of physical abuse and the covariates included in this analysis was based on a well-established internationally standardized instrument (13) which has been validated in the Albanian context before the implementation of the first round of HBSC in 2009–10. On the face of it, there is no reason to assume a differential reporting of lifetime physical abuse between schoolchildren pertinent to different categories of sociodemographic characteristics or lifestyle factors. Yet, the possibility of information bias cannot be entirely excluded, particularly for sensitive issues such as the experienced physical abuse and/or the witnessed domestic violence. Of note, sexual and emotional abuse which are more likely to affect the quality of life were not included in this study. Also, physical abuse at school premises was not assessed in the current study, which is another limitation. In addition, several lifestyle characteristics may have been under-reported including smoking, alcohol consumption or drug use. Furthermore, cross-sectional associations are not assumed to be causal and, therefore, the observed relationships between lifetime physical abuse and covariates should be interpreted with caution.

## Conclusion

Regardless of potential limitations, our study provides novel evidence about selected independent correlates of lifetime physical abuse among schoolchildren aged 15 years in Albania, a country still embedded in an everlasting transition which is associated with tremendous changes in family structure, community links and societal norms and values.

Health care practitioners and other professionals in Albania and elsewhere who are involved in child protection and child care should consider screening of children for substance use (including especially smoking and alcohol consumption), which may serve as important proxy indicators providing useful clues for child physical abuse. In addition, child care practitioners should be aware of the importance of assessing mental health and life satisfaction in general, which also provide valuable clues about the experiencing of child abuse.

In conclusion, policymakers and decision-makers in Albania and in other countries should be aware of the negative impact of child abuse and implement prompt and effective programs for control and prevention of all forms of child maltreatment. Interdisciplinary approaches should be employed for implementation of national programs in order to achieve success in prevention of child abuse and its negative health consequences (6). The current study conducted in Albania contributes to the international literature providing important evidence on selected independent risk factors of child physical abuse. This information will be useful for researchers, policymakers and decision-makers, but it will be especially valuable for practitioners involved in child protection and other child care services.

## Data Availability Statement

The raw data supporting the conclusions of this article will be made available by the authors, without undue reservation.

## Ethics Statement

The studies involving human participants were reviewed and approved by the Scientific Committee of the national Institute of Public Health in Albania (in 2017) and the data collection process was also confirmed by the Ministry of Education, Sport and Youth (in 2018). Written informed consent from the participants' legal guardian/next of kin was not required to participate in this study in accordance with the national legislation and the institutional requirements. The ethics committee has waived the requirement for written informed consent to participate from the participants' legal guardians in line with the guidelines and recommendations envisaged in the HBSC protocol.

## Author Contributions

GQ, QD, and GB contributed to the study conceptualization and design, analysis and interpretation of the data, and wrote the first draft of the article. IM, XD, LK, and ER commented comprehensively on the manuscript. All authors have read and approved the submitted manuscript.

## Conflict of Interest

The authors declare that the research was conducted in the absence of any commercial or financial relationships that could be construed as a potential conflict of interest. The reviewer DC declared a past collaboration with one of the authors GB to the handling editor.

## Publisher's Note

All claims expressed in this article are solely those of the authors and do not necessarily represent those of their affiliated organizations, or those of the publisher, the editors and the reviewers. Any product that may be evaluated in this article, or claim that may be made by its manufacturer, is not guaranteed or endorsed by the publisher.
